# Ultrasensitive and Rapid Visual Detection of *Escherichia coli* O157:H7 Based on RAA-CRISPR/Cas12a System

**DOI:** 10.3390/bios13060659

**Published:** 2023-06-16

**Authors:** Lishan Zhu, Zhenda Liang, Yongtao Xu, Zhiquan Chen, Jiasi Wang, Li Zhou

**Affiliations:** 1 Institute of Environmental Research at Greater Bay Area, Key Laboratory for Water Quality and Conservation of the Pearl River Delta, Ministry of Education, Guangzhou University, Guangzhou 510006, China; 2112004088@e.gzhu.edu.cn (L.Z.); lzd1996@e.gzhu.edu.cn (Z.L.); 2112104023@e.gzhu.edu.cn (Y.X.); 2112104033@e.gzhu.edu.cn (Z.C.); 2 Guangdong Provincial Key Laboratory of Sensor Technology and Biomedical Instrument, School of Biomedical Engineering, Shenzhen Campus of Sun Yat-Sen University, Shenzhen 518107, China

**Keywords:** CRISPR/Cas12a, RAA, *E. coli* O157:H7, on-site, visual detection, facile

## Abstract

*Escherichia coli* (*E. coli*) O157:H7 is a major foodborne and waterborne pathogen that can threaten human health. Due to its high toxicity at low concentrations, it is crucial to establish a time-saving and highly sensitive in situ detection method. Herein, we developed a rapid, ultrasensitive, and visualized method for detecting *E. coli* O157:H7 based on a combination of Recombinase-Aided Amplification (RAA) and CRISPR/Cas12a technology. The CRISPR/Cas12a-based system was pre-amplified using the RAA method, which showed high sensitivity and enabled detecting as low as ~1 CFU/mL (fluorescence method) and 1 × 10^2^ CFU/mL (lateral flow assay) of *E. coli* O157:H7, which was much lower than the detection limit of the traditional real-time PCR technology (10^3^ CFU/mL) and ELISA (10^4^~10^7^ CFU/mL). In addition, we demonstrated that this method still has good applicability in practical samples by simulating the detection in real milk and drinking water samples. Importantly, our RAA-CRISPR/Cas12a detection system could complete the overall process (including extraction, amplification, and detection) within 55 min under optimized conditions, which is faster than most other reported sensors, which take several hours to several days. The signal readout could also be visualized by fluorescence generated with a handheld UV lamp or a naked-eye-detected lateral flow assay depending on the DNA reporters used. Because of the advantages of being fast, having high sensitivity, and not requiring sophisticated equipment, this method has a promising application prospect for in situ detection of trace amounts of pathogens.

## 1. Introduction

Since being identified as a human pathogen in 1982, the zoonotic life-threatening Shiga-toxin-producing *E. coli* has become a serious public health concern [1,2]. Among the known serotypes of *E. coli* O157:H7 is a notorious pathogen confirmed in outbreaks of illness in many countries such as Canada, the United Kingdom, Ireland, the United States, and China [1]. As a foodborne and waterborne pathogen, *E. coli* O157:H7 is transmitted to humans primarily through contaminated food and water and direct contact with infected humans or animals [3,4,5]. Its infection can cause bloody diarrhea, hemorrhagic colitis, and hemolytic uremic syndrome [4,5,6,7]. It is estimated that more than two million *E. coli* O157:H7 infections occur worldwide each year. A low dose (10–100 cells) of *E. coli* O157:H7 infection can lead to disease or even death, especially in immunocompromised individuals. Furthermore, a long detection time of *E. coli* O157:H7 may result in antibiotic use without bacterial testing, potentially leading to the growth of antibiotic resistance [8,9,10]. Therefore, an ultrasensitive, rapid, and specific detection method is particularly necessary to identify *E. coli* O157:H7 in food and drinking water.

Currently, detection methods for *E. coli* O157:H7 include both traditional culture-based methods, immunoassay approaches, and molecular detection methods [3,11,12,13,14,15]. The traditional culture-based method mainly involves bacterial culture, colony morphology observation, and special biochemical characteristics’ measurement. Although this method is stable and reliable, the process is relatively tedious, time-consuming (~7 days for identification), and has a heavy workload, which makes rapid prevention and control of pathogenic bacteria difficult. The immunological detection methods include the enzyme-linked immunosorbent assay (ELISA), the Immunomagnetic separation (IMS), the immunofluorescence test (IFT), and immunochromatographic test (ICT) [16,17,18,19]. However, they may have several drawbacks such as high costs and low sensitivity. To address these issues, nucleic-acid-based methods, which have high specificity and a short operation time, have been exploited in recent years [20,21,22,23,24]. Representative methods include polymerase chain reaction (PCR), quantitative real-time PCR (qPCR), multiplex PCR, and gene chips [20,21,22,23,24]. However, these methods may require special instruments, complex steps, and well-trained technicians, which further result in practical inconvenience. Meanwhile, the inability to perform diagnostic point-of-care testing (POCT) hinders their extensive application [20]. RAA is a novel method that works under isothermal conditions, which shows tremendous potential for applications in the molecular diagnosis of POCT. Compared to traditional PCR, it has the advantages of a fast reaction time, no requirement of special equipment, and an optimal working temperature of 37 °C. This enables it to better meet the requirements of rapid and simple on-site pathogen detection. However, false-positive results sometimes can be generated due to cross-contamination and non-specific amplification, which hinder its wide application.

Recently, clustered regularly interspaced short palindromic repeats (CRISPR) have attracted extensive attention due to their potential application in gene editing, nucleic acid testing, transcriptional regulation, and gene therapy [25,26,27,28,29,30]. The CRISPR/Cas system, consisting of CRISPR and Cas proteins, was first discovered in prokaryotes as an immune defense system against the invasion of foreign viruses, phages, plasmids, and other genetic elements [31]. Furthermore, the proteins Cas12, Cas13, and Cas14 have sequence-specific recognition, endonuclease activity, and target-activated trans-cleavage activity guided by CRISPR RNA (crRNA). After specifically recognizing the target sequence, the Cas-crRNA complex can activate trans-cleavage activity to randomly degrade ssDNA or ssRNA, which can realize the tasks of recognition and signal transduction simultaneously. Among the many CRISPR/Cas systems, the CRISPR/Cas12a system has precise DNA targeting and cutting functions, which can be easily combined with various nucleic acid amplification technologies [32,33]. Several isothermal amplification technologies (IATs), such as loop-mediated isothermal amplification (LAMP) and recombinase polymerase amplification (RPA), were combined with CRISPR/Cas12a to detect pathogens [34,35,36,37,38,39]. For instance, following the outbreak of the SARS-CoV-2 pandemic, the LAMP assay was combined with the CRISPR/Cas system for sensitive detection of SARS-CoV-2 infection. Through combining a gold-nanoparticle-based visual assay with the LAMP-CRISPR/Cas12a system, our group further realized high-throughput visual detection of SARS-CoV-2 [40]. Pei et al. also developed a novel high-throughput RPA-CRISPR/Cas12a method for sensitively monitoring pathogenic *Staphylococcus aureus* in the water environment. These studies suggested that the combination of IATs and the CRISPR/Cas12a system enable improving the sensitivity and specificity, which is particularly suitable for the development of highly sensitive POCT technology.

In the present study, we aimed to achieve ultrasensitive and rapid detection of *E. coli* O157:H7 using the CRISPR/Cas12a system combined with RAA, using the O157 antigen gene cluster *rfbE* as the target. In the established system, the detection process could be completed within 51–55 min by optimizing the whole processes including DNA extraction, RAA amplification, and the CRISPR/Cas cutting step. Test strips and ultraviolet lamps were used to visualize the results directly. Since the technology does not require complex equipment, the cost was greatly reduced. Moreover, the RAA amplification technique combined with CRISPR/Cas12a technology greatly improved the sensitivity of the assay. This study provides a rapid, ultrasensitive, and non-quantitative method for in situ detection of pathogenic microorganisms existing in the food and water environments.

## 2. Materials and Methods

### 2.1. Materials and Apparatus

The Ezup Column Bacterial Genomic DNA Extraction Kit was purchased from Sangon Biotech Co., Ltd. (Shanghai, China). The RAA nucleic Acid Amplification Kit was bought from Jiangsu Qitian Gene Biotechnology Co., Ltd. (Jiangsu, China). Oligonucleotides were synthesized by Sangon Biotech Co., Ltd. (Shanghai, China). The oligonucleotide sequences used in this study are listed in Table S1. Cas12a was purchased from New England Biolabs. The Cas12/13 special nucleic acid test strip and 1 × Buffer were purchased from Guangzhou Bio-lifesci Co., Ltd. (Guangzhou, China). DEPC water (DNase-/RNase-free) and the Ribonuclease Inhibitor (RNase Inhibitor) were obtained from Beyotime Institute of Biotechnology (Shanghai, China). Tris-saturated Phenol was purchased from Beijing Solarbio Science & Technology Co., Ltd. (Beijing, China). Gelstain was bought from TransGen Biotech Co., Ltd. (Beijing, China). All reagents were used as received and without further purification.

The oligonucleotide annealing process and RAA reaction were carried out by T100 Thermal Cycler PCR (from Bio-Rad, Tokyo, Japan). Gel imaging was performed using the Tanon 1200 imaging system. The fluorescence spectra were measured by the LightCycler96 qPCR (from Roche, Basel, Switzerland).

### 2.2. Bacterial Strains and Culture Conditions

The bacterial strains used in this study are listed in Table S2. All strains were cultured in a culture bottle containing Luria–Bertani (LB) broth and kept in a constant temperature incubator at 37 °C.

### 2.3. Bacterial DNA Extraction

The DNA of all bacterial strains was extracted using the Ezup Column Bacterial Genomic DNA Extraction Kit according to the manufacturer’s instructions. The extracted DNA was stored at −20 °C or immediately used for the experiments.

### 2.4. Isothermal Amplification

Isothermal amplification reactions were performed according to the instructions of the RAA nucleic Acid Amplification Kit. Each reaction contained one tube of reaction unit, 25 μL of Buffer V, 2 μL of the forward and reverse primers (10 μM), 2.5 μL of magnesium acetate (MgAc), and the DNA template. Purified water was added to bring the total volume of the mixture to 50 μL. The mixture reacted at 37 °C for 20 min. After the reaction, 50 μL of phenol/chloroform (1:1) was added to each reaction tube. The amplified products were detected by 1.5–2.0% agarose gel electrophoresis, stained with Gelstain, and visualized under an automatic gel imaging system. The primer sequences for specific *rfbE* were designed based on the published work [33].

### 2.5. CRISPR/Cas12a Fluorescence Assay

In the CRISPR/Cas12a detection system, 400 nM Cas12a (Cpf1), 300 nM crRNA, and 1× Buffer were mixed and incubated for 5 min on ice. Then, 1 μL of the RNase Inhibitor (40 U/μL) and 400 nM ssDNA-FQ (FAM-ssDNA-BHQ1) reporter were added. Finally, 1 μL of amplified product was added. The 20 μL reaction system was placed at 37 °C for 40 min in a LightCycler96 machine, with fluorescence measurements taken every 1 min (λex: 492 nm; λem: 518 nm). The sequences of crRNA and reporters are listed in Table S1.

### 2.6. CRISPR/Cas12a-LFA Assay

The nucleic acid test strip consisted of an absorption pad, an interpretation area, and a binding pad. Anti-FAM antibody-conjugated Au NPs were embedded in the binding pad; streptavidin was embedded in the C line, and the secondary antibody was embedded in the T line in the interpretation area. In the LFA assay, reporter ssDNA probes were labeled with FAM and biotin at the 5′ and 3′ ends, respectively. The Cas12a-mediated cleavage assay contained 400 nM Cas12a, 300 nM crRNA, 400 nM ssDNA-FB reporter, and a certain amount of Buffer with 1 μL of substrate dsDNA in a 20 μL reaction volume. After reaction at 37 °C for 40 min, ultrapure water was added to bring the volume of the reaction to 50 μL. A test strip was placed into the solution for signal readout. The results can be directly visualized or photographed within 10 min based on the color of the test strip. The purified water was used as a negative control for the experiment.

### 2.7. Sensitivity and Specificity Tests

The optimized CRISPR/Cas12a system and isothermal amplification time were used to evaluate the sensitivity and specificity of the system. Then, 10-fold serial dilutions of *E. coli* O157:H7 bacterial concentrations were used for sensitivity detection (the initial concentration of the bacterial solution was 7 × 10^6^ CFU/mL), and DNase-/RNase-free DEPC water was used as a non-template control (NTC). All experiments were performed in triplicate.

Additionally, one strain of target bacteria and six other strains of non-target bacteria were selected to validate the specificity of RAA-CRISPR/Cas12a (Table S2). To save reagents, the CRISPR /Cas12a reaction system was halved for detection. All experiments were repeated three times.

### 2.8. Detection of E. coli O157:H7 in Milk and Drinking Water

Skim milk and drinking water were used to evaluate the applicability of the system. The samples (including drinking water and skim milk) were purchased from a local market in Guangzhou without further treatment. *E. coli* O157:H7 was spiked in skim milk and drinking water, respectively, to achieve concentrations ranging from 10^0^ to 10^6^ CFU/mL using 10-fold serial dilutions (the initial concentration of the bacterial solution was 7 × 10^6^ CFU/mL). DEPC water was used as a negative control. After that, DNA extraction and RAA amplification were performed following the methods described above. The resulting solution was further used for the CRISPR/Cas12a system. To save reagents, the CRISPR /Cas12a reaction system was halved for detection. All experiments were repeated three times.

### 2.9. Optimizing the Time Required for the Entire Detection Process

The bacteria DNA extraction, amplification, CRISPR/Cas12a cutting, and detection process were performed as described above, but with shorter reaction times of 25, 10, 15, and 5 min for each step.

## 3. Results and Discussion

### 3.1. CRISPR/Cas12a-Based E. coli O157:H7 Genomic DNA Detection System

**Figure 1:**
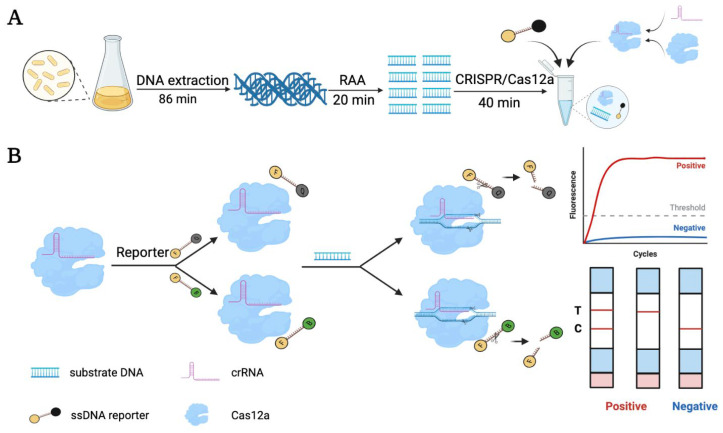
Schematic illustration of detecting the *E. coli* O157:H7 using the RAA-CRISPR/Cas12a system. (**A**) Workflow for CRISPR/Cas12a-mediated detection of *E. coli* O157:H7. It included genomic DNA extraction, RAA preamplification, and Cas12a/crRNA cleavage assay. The times required for each step are based on the Kit’s instructions or previous studies. (**B**) The detection principle and signal readout process of CRISPR/Cas12a. The nucleic acid test strip comprises an absorption pad, an interpretation area, and a binding pad (Figure S1). The binding pad contains embedded Au NPs; the C line contains embedded streptavidin; the T line in the interpretation area contains embedded secondary antibody for signal readout. A red band appearing on the T line can be judged as a positive result.

An RAA-CRISPR/Cas12a detecting system, which consisted of the Cas12a protein, specific crRNAs, ssDNA probes, and target dsDNA, was successfully established for rapid, specific, and sensitive detection of *E. coli* O157:H7. Utilizing the programmability of the CRISPR/Cas12a system, crRNA is designed according to the principle of base pairing with the target sequence, which is partially complementary to the target sequence. Combining the nucleic acid amplification method with CRISPR/Cas12a, the detection sensitivity of the target nucleic acid was doubled (Figure 1A). First, Cas12a and crRNA were incubated to form functional complexes. After that, the crRNA guided the Cas12a nuclease to specifically recognize and cleave the target dsDNA, and then, the enzyme’s trans collateral activity was immediately activated to randomly shear and degrade the ssDNA probe. Based on this principle, the fluorescence-quenched ssDNA probe was used as a fluorescence reporter. After the DNase activity of the Cas12a-crRNA complex was activated, the fluorescence reporter was simultaneously cleaved, leading to the restoring of the fluorescence signal, which can be used for target detection. Besides, a lateral flow readout based on a fluorescein (FAM) and biotin dual-modified ssDNA reporter was also constructed because of their low cost and ease of operation. With this type of ssDNA reporter, test results can be read out directly according to the color of the strip (Figure 1B). As shown in Figure S1, the strip was composed of a binding pad, an interpretation area, and an absorbing pad. The binding pad was coated with anti-FAM antibody-conjugated Au NPs, which can specifically bind FAM and exhibit the band signal on the strip. Streptavidin and the secondary antibody were coated in the C and T lines of the interpretation area, respectively. The cleaved ssDNA reporter by CRISPR/Cas12a can migrate along the strip until it is captured by the secondary antibody, which leads to a red T line. The un-cleaved reporter can bind to the anti-FAM antibody-conjugated Au NPs and further be captured by the streptavidin to help verify the accuracy of the test results (Figure S1). Notably, the entire strip test took less than 10 min.

### 3.2. Optimization of the RAA-CRISPR/Cas12a Reaction System

**Figure 2:**
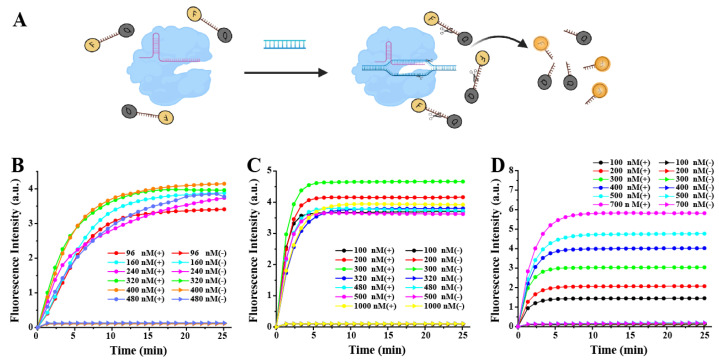
Optimization of the RAA-CRISPR/Cas12a reaction system. (**A**) A schematic illustration of the RAA-CRISPR/Cas12a-FQ detector. Real-time fluorescence detection assay using the CRISPR/Cas12a-FQ detector with different concentrations of (**B**) Cas12a (crRNA 480 nM and ssDNA-FQ reporter 400 nM), (**C**) crRNA (Cas12a 400 nM and ssDNA-FQ reporter 400 nM), and (**D**) ssDNA-FQ reporter (Cas12a 400 nM and crRNA 480 nM).

The feasibility of using the CRISPR/Cas12a system for bacterial target sequence detection was evaluated (Figure 2A). The *Escherichia coli rfbE* gene was selected as the target gene. To improve the sensitivity and specificity of the method, we investigated the optimal concentration of the crRNA, Cas12a, and ssDNA reporter in the reaction system, as the cleavage activity was directly related to their concentration. The concentration of target DNA used in the experiment was ~5 nM. In the 20 μL reaction system, the fluorescence value reached the highest when the concentrations of the Cas12a and crRNA were 400 nM and 300 nM, respectively (Figure 2B,C). The higher amount of the crRNA or Cas12a probably affected the efficiency of the trans-cleavage of CRISPR/Cas12a [41,42]. The concentration of the ssDNA reporter was also critical in the CRISPR/Cas12a detection system. As shown in Figure 2D, we found that, with the increase of the probes’ concentration, the fluorescence intensity also increased. In order to better distinguish negative samples without wasting reagents, 400 nM ssDNA reporter was used for the subsequent experiment. To understand the effect of the component ratios on the assay sensitivity, different Cas12a/crRNA ratios were used in the CRISPR/Cas12a assay. It was found that the optimal Cas12a/crRNA ratio was 4:3, which also agreed with previous studies (Figure S2). A suitable amplification time of genomic DNA is an important parameter for CRISPR/Cas12a analysis. Therefore, different reaction times were used for the target DNA amplification, and the optimal amplification time found was 20 min (Figure S3).

### 3.3. Detection Sensitivity of the RAA-CRISPR/Cas12a System

**Figure 3:**
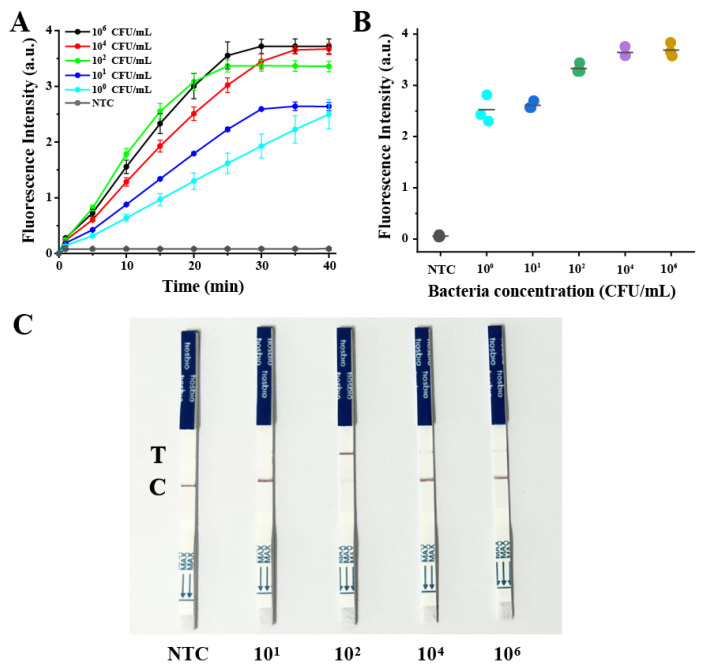
Evaluation of the detection sensitivity of the RAA-CRISPR/Cas12a system. (**A**) Real-time fluorescence detection assay using the CRISPR/Cas12a-FQ detector. (**B**) Fluorescence generated with the different concentrations of *E. coli* O157:H7. (**C**) Results of LFAs treated with different concentrations of *E. coli* O157:H7. NTC: no-template control.

Under the optimized reaction conditions, the detection sensitivity of the system toward the *E. coli* O157:H7 samples was estimated. As shown in Figure 3, compared with the negative control, the fluorescence intensity increased significantly with *E. coli* O157:H7 ranging from 10^0^ CFU/mL to 10^6^ CFU/mL. This agreed well with the gel electrophoresis pattern of the target DNA amplified by RAA (Figure S4). These results demonstrated that the RAA-CRISPR/Cas12a method developed here had high detecting sensitivity. Even samples with a concentration of ~1 CFU/mL produced significant signals, which is much lower than the detection limit of the traditional real-time PCR technology (10^3^ CFU/mL), ELISA (10^4^~10^7^ CFU/mL), and some recently developed methods (Table S3) [43,44]. The excellent performance of this method can be attributed to the combination of nucleic acid amplification technology and the CRISPR/Cas12a system and the optimized reaction conditions, which enabled specific identification of the target genes and further efficient amplification of the signal. Although the experimental results did not show a good linear relationship between the *E. coli* O157:H7 concentration and fluorescence intensity, it can be very useful for the identification of trace *E. coli* O157:H7 infection. This phenomenon may be attributed to the fact that the developed system involves RAA amplification and CRISPR/Cas12a cutting. Achieving a linear response requires that the product generated through RAA amplification is proportional to the target concentration and that a linear correlation exists between the RAA product and the fluorescence signal from the CRISPR/Cas12a step. This can be difficult to achieve, and a similar non-linear relationship has been observed in some reported sensors that combine DNA amplification and the CRISPR/Cas system [40,45]. For naked-eye observation, the CRISPR/Cas12a-LFA assay was carried out using the FAM and biotin dual-modified ssDNA reporter. Significantly, a flow strip can generate a visible signal when the concentration of *E. coli* O157:H7 is as low as 1 × 10^2^ CFU/mL (Figure 3C). This indicates that the RAA-CRISPR/Cas12a method can realize rapid visual detection with high sensitivity.

### 3.4. Specificity of the CRISPR/Cas12a System for Detecting E. coli O157:H7

**Figure 4:**
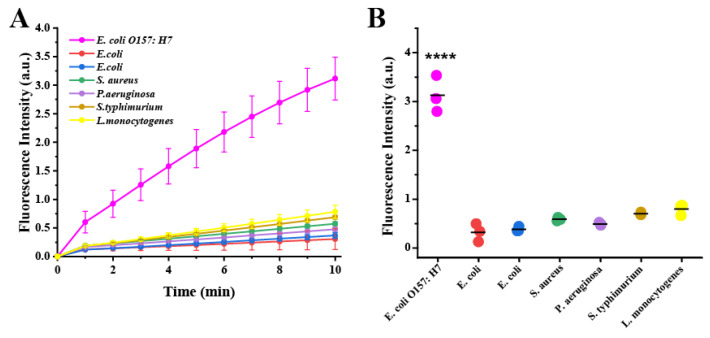
Specificity of the CRISPR/Cas12a system for detecting *E. coli* O157:H7. (**A**) Real-time fluorescence detection assay using the CRISPR/Cas12a-FQ detector for different bacterial strains. (**B**) Fluorescence generated from different bacterial strains. Numbers 1–7 represent *E. coli* O157:H7 (CICC 24187), *E. coli* (ATCC 25922), *E. coli* (ATCCBAA2452), *S. aureus* (CICC 10306), *P. aeruginosa* (CICC 21636), *S. typhimurium* (CICC 22956), and *L. monocytogenes* (CICC 21635), respectively. Significant differences between groups were tested with the independent samples *t*-test (**** represents *p* < 0.0001).

To verify the specificity of the CRISPR/Cas12a system, five different bacterial strains (*E. coli*, *S. aureus*, *P. aeruginosa*, *S. typhimurium*, *L. monocytogenes*) were selected, and the fluorescence signal was recorded. Compared with the target bacterial strain, the other non-target bacterial strains exhibited a low fluorescence signal, which was consistent with the results of gel electrophoresis (Figure 4 and Figure S5). The results confirmed the high specificity and good selectivity of this method. It should be noted that the specificity could be attributed to the recognition function of the Cas12a/crRNA complex.

### 3.5. The Application Potential of the RAA-CRISPR/Cas12a System

**Figure 5:**
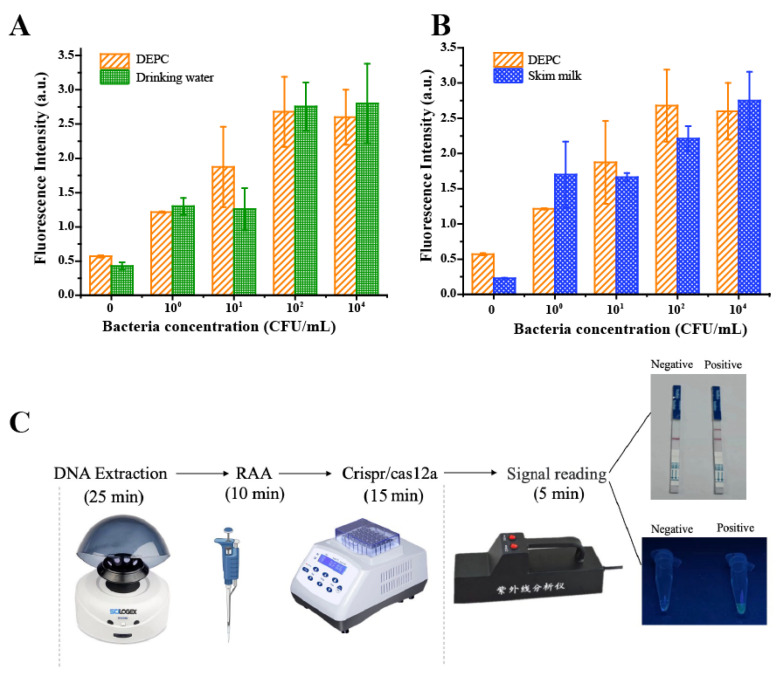
The application potential of the RAA-CRISPR/Cas12a system. The fluorescence signal against varying *E. coli* O157:H7 concentrations in drinking water (**A**) and skim milk (**B**). The fluorescence intensity of *E. coli* O157:H7 in DEPC water (DNase-/RNase-free) was also recorded for comparison. (**C**) The schematic of the time cost of the developed system for visualized detection.

Since the presented method demonstrated high sensitivity and specificity, the potential application of the approach in real samples including skim milk and drinking water was further investigated. The standard addition of different concentrations of *E. coli* O157:H7 (10^4^, 10^2^, 10^1^, and 10^0^ CFU/mL) in practical samples was carried out, followed by sample detection with the RAA-CRISPR/Cas12a method. As shown in Figure 5 and Table S4, the method displayed good detecting sensitivity in both the milk and drinking water samples, demonstrating the applicability of the method.

In order to achieve rapid, sensitive, and simple in situ detection of *E. coli* O157:H7, the entire detection process was optimized (Figure 5C). The three processes, including DNA extraction, RAA amplification, and CRISPR reaction can be performed with just two simple, portable instruments: a handheld centrifuge and a metal bath. In terms of signal reading, the test results can be visualized with a handheld UV analyzer and test strip by adding different ssDNA reporters, which allows the results to be displayed more intuitively without complicated instruments and data processing. The cost coming from the strips can be solved by mass production. Thus, we expect future tests to cost only a few dollars. Besides, on the premise of being distinguishable from the negative control, the duration of the entire test process can be shortened to less than 55 min, which improved the efficiency of the test. Our proposed method provides a rapid, simple in situ assay for the detection of *E. coli* O157:H7 without quantification.

## 4. Conclusions

In this study, a rapid and ultrasensitive method for the detection of *E. coli* O157:H7 was developed based on the RAA-CRISPR/Cas12a system. By combining the CRISPR/Cas12a system with isothermal amplification techniques, *E. coli* O157:H7 concentrations as low as ~1 CFU/mL (fluorescence method) and 1 × 10^2^ CFU/mL (lateral flow assay) can still be detected with significant accuracy, which was superior to most previously reported methods. Moreover, this method achieved high specificity among different strains and showed good performance in practical sample analysis. Importantly, we optimized the whole reaction process, allowing the detection process to be completed within 55 min, which was faster than most other reported sensors, which take several hours to several days. Signal visualization could be achieved by a handheld UV lamp or lateral flow strips, which facilitates the in situ detection of *E. coli* O157:H7 without complex instruments. Overall, our study provides a simple and facile method for the evaluation of *E. coli* O157:H7 contamination, which can also be used for the detection of other pathogens. This method has broad application prospects in the fields of food safety, clinical diagnosis, and environmental science.

## Data Availability

The experimental data are contained within the article.

## Supplementary Materials

The following Supporting Information can be downloaded at: https://www.mdpi.com/article/10.3390/bios13060659/s1, Figure S1: Principle of visual detection with LFA; Table S1: Sequences used in this study; Figure S2: Effects of different Cas12a/crRNA ratios on fluorescence intensity; Table S2: Verification of CRISPR/Cas12a specificity; Figure S3: Effect of different amplification times of RAA on fluorescence intensity; Table S3: Detection limit of *E. coli* O157:H7 with different methods [32,33,43,46,47,48,49]; Figure S4: The gel electrophoresis result of RAA for different bacterial concentrations; Table S4: Detection of *E. coli* O157:H7 in milk and drinking water samples using fluorescence signal; Figure S5: The gel electrophoresis result of RAA for different bacterial strains.
