# Supplementary material for: Ultrasensitive and Rapid Visual Detection of Escherichia coli O157:H7 Based on RAA-CRISPR/Cas12a System
Source: Biosensors (Basel). 2023 Jun 16;13(6):659. doi: 10.3390/bios13060659 (PMC10295930; doi:10.3390/bios13060659)
Supplement: Supplementary file 1 [file biosensors-13-00659-s001.zip › biosensors-2417211-supplementary.pdf]

biosensors MDPI

Supplementary Materials

Ultrasensitive and Rapid Visual Detection of *Escherichia coli* O157:H7 Based on RAA–CRISPR/Cas12a System

Lishan Zhu[1], Zhenda Liang[1], Yongtao Xu, Zhiquan Chen, Jiasi Wang[2,*] and Li Zhou[1,*]

1. Institute of Environmental Research at Greater Bay Area, Key Laboratory for Water Quality and Conservation of the Pearl River Delta, Ministry of Education, Guangzhou University, Guangzhou, 510006, P. R. China

2. Guangdong Provincial Key Laboratory of Sensor Technology and Biomedical Instrument, School of Biomedical Engineering, Shenzhen Campus of Sun Yat-sen University, Shenzhen, 518107, China.

Corresponding author: wangjs8@mail.sysu.edu.cn (J. Wang), zhoul@gzhu.edu.cn (L. Zhou).

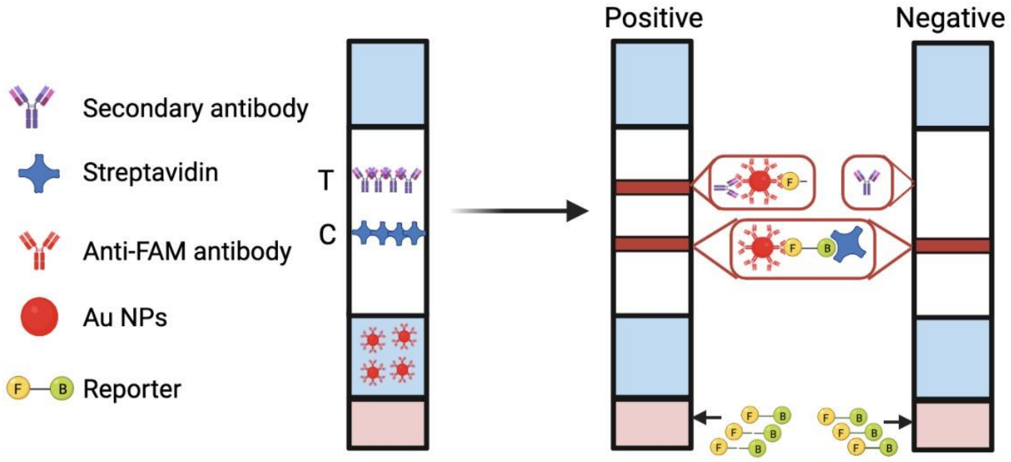

Fig. S1. Principle of visual detection with LFA.

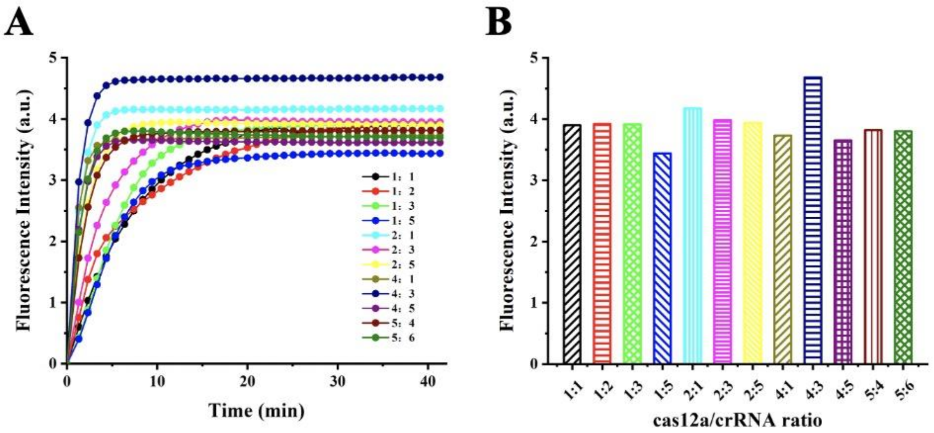

Fig. S2. Effects of different Cas12a/crRNA ratios on fluorescence intensity. A) Real-time fluorescence detection assay using the CRISPR/Cas12a-FQ detector for different Cas12a/crRNA ratios. B) Fluorescence generated from different Cas12a/crRNA ratios.

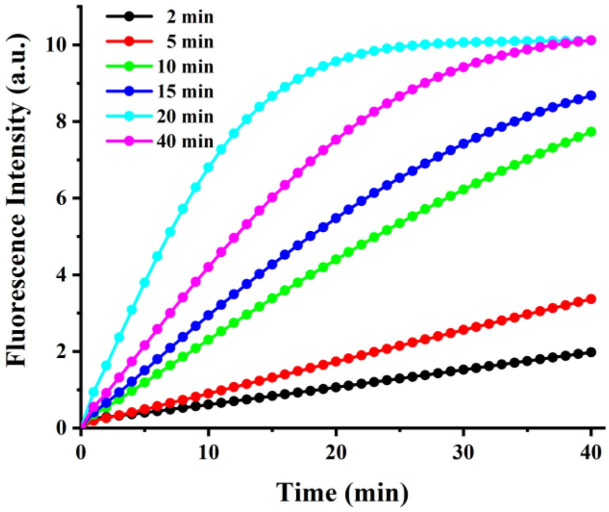

Fig. S3. Effect of different amplification times of RAA on fluorescence intensity.

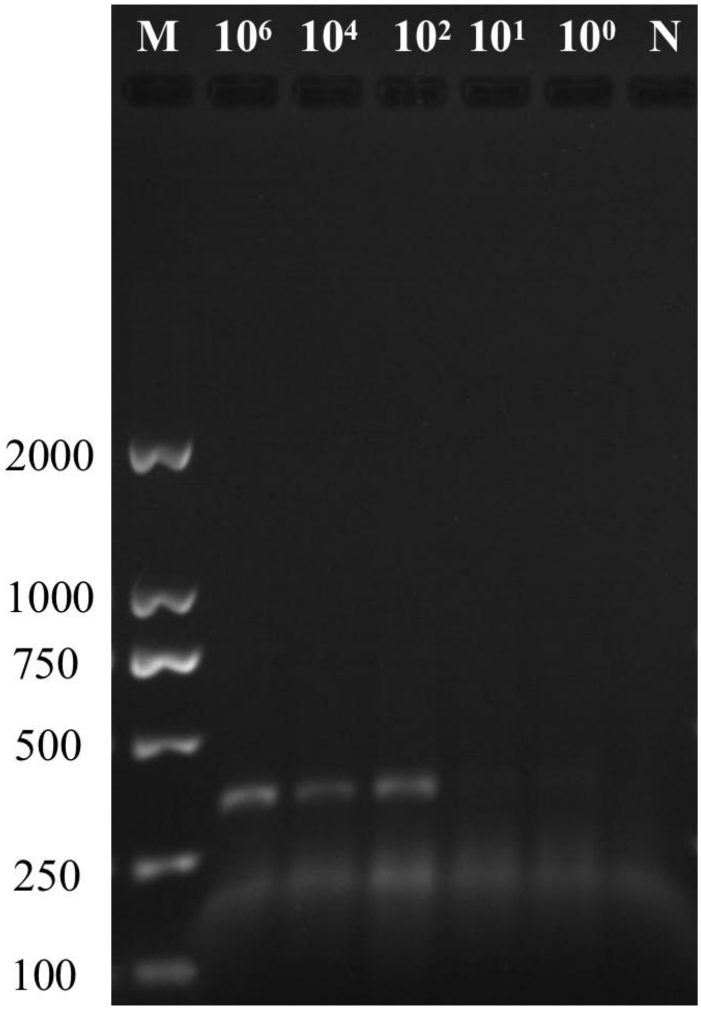

Fig. S4. The gel electrophoresis result of RAA for different bacterial concentration.

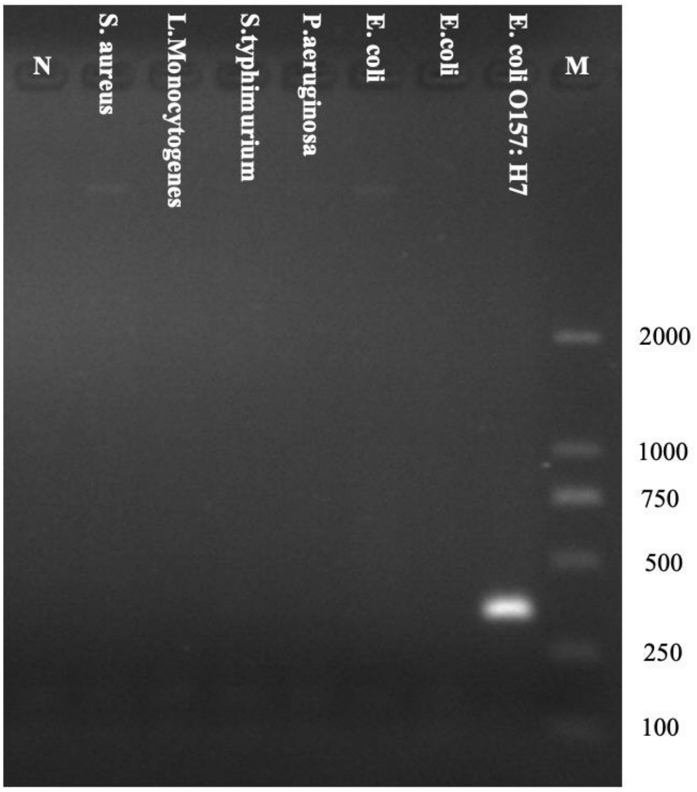

Fig. S5. The gel electrophoresis result of RAA for different bacterial strains.

Table S1. Sequences used in this study [1]

Method	Primer's name	Sequence (5′ to 3′)
RAA	rfbE-F1	AACATATCGATAGACAGTTAAATATAAGAG
	rfbE-R1	TTTGCCAAGTTTCATTATCTGAATCAACG
CRISPR/Cas12a	LbCas12a crRNA	GGGUAAUUUCUACUAAGUGUAGAUCCAACCGUCAUUGACAGGAA
	ssDNA-FB reporter	6-FAM-TTTTTT-Biotin
	ssDNA-FQ reporter	6-FAM-TTTTTT-BHQ1

Table S2. Verification of CRISPR/Cas12a specificity

No.	Bacterial species	Strains	CRISPR/Cas12 system results
1	*E. coli* O157: H7	CICC 24187	+
2	*E. coli*	ATCC25922	-
3	*E. coli*	ATCCBAA2452	-
4	*S. aureus*	CICC10306	-
5	*P. aeruginosa*	CICC21636	-
6	*S. typhimurium*	CICC22956	-
7	*L. monocytogenes*	CICC21635	-

CICC: China Center of Industrial Culture Collection; ATCC: American Type Culture Collection. “+/-” indicates positive and negative results.

Table S3 Detection limit of E. coli O157:H7 with different method

No.	Method	LOD (CFU/mL)	Time (min)	Ref.
1	RPA-CRISPR/Cas12a	6.5×10^4	> 100	[1]
2	ELISA	1×10^4	> 480	[2]
3	p-ELISA	1×10^4	< 180	[3]
4	I-IMS-FLFI	2.39×10^2	90	[4]
5	ICT	1.8<br>(Minimum concentration)	/	[5]
6	RT-PCR	6.4×10^3	/	[6]
7	A One-Pot Toolbox	1	< 50	[7]
8	RAA-CRISPR/Cas12a	1<br>(Minimum concentration)	< 55	This work

Table S4 Detection of *E. coli* O157:H7 in milk and drinking water samples using fluorescence signal

samples	spiked (CFU/mL)	fluorescence	RSD (n=3)
DEPC	0	0.57	0.016
	1.0×10^{0}	1.21	0.0084
	1.0×10^{1}	1.87	0.58
	1.0×10^{2}	2.68	0.51
	1.0×10^{4}	2.61	0.42
Drinking water	0	0.43	0.052
	1.0×10^{0}	1.31	0.12
	1.0×10^{1}	1.26	0.31
	1.0×10^{2}	2.76	0.35
	1.0×10^{4}	2.83	0.58
Skim milk	0	0.23	0.0069
	1.0×10^{0}	1.70	0.47
	1.0×10^{1}	1.67	0.059
	1.0×10^{2}	2.21	0.18
	1.0×10^{4}	2.75	0.41

References

1. Wang, S.; Fan, Y.; Feng, Z.; Song, M.; Li, Q.; Jiang, B.; Qin, F.; Liu, H.; Lan, L.; Yang, M. Rapid Nucleic Acid Detection of Escherichia Coli O157:H7 Based on CRISPR/Cas12a System. Food Control 2021, 130, 108194.
2. Feng, M.; Yong, Q.; Wang, W.; Kuang, H.; Wang, L.; Xu, C. Development of a Monoclonal Antibody-Based ELISA to Detect Escherichia Coli O157:H7. Food and Agricultural Immunology 2013, 24, 481–487.
3. Pang, B.; Zhao, C.; Li, L.; Song, X.; Xu, K.; Wang, J.; Liu, Y.; Fu, K.; Bao, H.; Song, D.; et al. Development of a Low-Cost Paper-Based ELISA Method for Rapid Escherichia Coli O157:H7 Detection. Analytical Biochemistry 2018, 542, 58–62.
4. Shen, Z.; Hou, N.; Jin, M.; Qiu, Z.; Wang, J.; Zhang, B.; Wang, X.; Wang, J.; Zhou, D.; Li, J. A Novel Enzyme-Linked Immunosorbent Assay for Detection of Escherichia Coli O157:H7 Using Immunomagnetic and Beacon Gold Nanoparticles. Gut Pathogens 2014, 6, 14.
5. Jung, B.Y.; Jung, S.C.; Kweon, C.H. Development of a Rapid Immunochromatographic Strip for Detection of Escherichia Coli O157. Journal of Food Protection 2005, 68, 2140–2143.
6. Ibekwe, A.M.; Watt, P.M.; Grieve, C.M.; Sharma, V.K.; Lyons, S.R. Multiplex Fluorogenic Real-Time

PCR for Detection and Quantification of Escherichia Coli O157:H7 in Dairy Wastewater Wetlands. Applied and Environmental Microbiology 2002, 68, 4853–4862.

7. Wang, Y.; Ke, Y.; Liu, W.; Sun, Y.; Ding, X. A One-Pot Toolbox Based on Cas12a/CrRNA Enables Rapid Foodborne Pathogen Detection at Attomolar Level. Acs Sensors 2020, 5, 1427–1435.
